# Practicalities of using an adaptive design for decision making within the optima trial: optimal personalized treatment of early breast cancer using multi-parameter tests

**DOI:** 10.1186/1745-6215-16-S2-P212

**Published:** 2015-11-16

**Authors:** Janet Dunn, Andrea Marshall, Amy Campbell, David Cameron, Helena Earl, Iain Macpherson, Christopher Poole, Daniel Rea, Adele Francis, Victoria Harmer, Adrienne Morgan, Nigel Stallard, Andreas Makris, Luke Hughes-Davies, Robert Stein

**Affiliations:** 1 University of Warwick, Coventry, UK; 2 University of Edinburgh, Edinburgh, UK; 3 University of Cambridge Dept. of Oncology, Cambridge, UK; 4 NIHR Cambridge Biomedical Research Centre, Cambridge, UK; 5 Beatson West of Scotland Cancer Centre, Glasgow, UK; 6 University Hospital Coventry, Coventry, UK; 7 University of Birmingham, Birmingham, UK; 8 University Hospitals Birmingham NHS Foundation Trust, Birmingham, UK; 9 Imperial College Healthcare Trust, London, UK; 10 Independent Cancer Patients’ Voice, London, UK; 11 Mount Vernon Hospital, Northwood, UK; 12 Cambridge University Hospitals NHS Foundation Trust, Cambridge, UK; 13 University College London Hospital, London, UK; 14 UCL NIHR Biomedical Research Centre, London, UK

## Background

Multi-parameter gene expression assays (MPA) to aid selection of chemotherapy in hormone-sensitive early breast cancer have not been prospectively validated. This field of personalised medicine is rapidly evolving. There is currently no “best test”. OPTIMA is an adaptive trial of MPA-based chemotherapy assignment in a largely node-positive breast cancer population.

## Methods and results

OPTIMA prelim, the feasibility phase, recruited 302 patients to establish acceptability of the trial to patients and clinicians and evaluate the performance of MPAs to identify a suitable test(s) to be used in the main efficacy trial. Ethical approval was also gained for an additional 200 patient roll through before the opening of the main trial. The logistics of this design and practicalities of rolling through will be discussed. Whilst this design was efficient and minimises bureaucracy, the delays encountered with this type of design stem from the need to produce pilot results quickly and for funding bodies to make quick decisions about the feasibility of the main trial.

## Conclusions

The success of OPTIMA not only relies on the integration of a multi-disciplinary team of methodologists, clinical experts and patients at all stages of the trial, but on its adaptive design. The complexities of using such methodology and decision-making to roll forward into a main trial are challenging but provide the most efficient use of patients and costs.

## Funding

Project funded by the NIHR HTA Programme (10/34/01). Views expressed are those of the authors and not those of the HTA programme, NIHR, NHS or the DoH.

